# Persistent maternal mental health and child’s behavioural, academic, and educational outcomes: evidence from national longitudinal study

**DOI:** 10.1093/pubmed/fdag032

**Published:** 2026-04-25

**Authors:** Minnat Seema, Clifford Afoakwah, Joshua Byrnes

**Affiliations:** Centre for Applied Health Economics (CAHE), Griffith University, 170 Kessels Rd, Nathan, Brisbane 4111, Queensland, Australia; Australian Centre for Health Services Innovation, School of Public Health and Social Work, Queensland University of Technology, Brisbane 4000, Australia; Jamieson Trauma Institute, Metro North Health, Brisbane 4006, Australia; Centre for Applied Health Economics (CAHE), Griffith University, 170 Kessels Rd, Nathan, Brisbane 4111, Queensland, Australia

**Keywords:** maternal mental health, psychological distress, child development, educational outcomes, longitudinal study

## Abstract

**Background:**

Maternal mental health is an important determinant of child development, yet most evidence relies on short exposure windows or single-wave measures. Less is known about how long-run maternal mental health relates to child outcomes when child development data are observed intermittently.

**Methods:**

Using nationally representative longitudinal household panel data, we examined associations between maternal mental health and child behavioural outcomes, academic performance, and expectations of future university participation. Child outcomes were observed in three survey waves, while maternal mental health was measured repeatedly over a longer period. Maternal mental health was operationalized as long-run averages of general mental health and psychological distress. Ordinary least squares models were estimated with adjustment for child, maternal, and household characteristics, and standard errors were clustered at the mother level.

**Results:**

Higher maternal psychological distress was associated with poorer academic performance and less favourable behavioural and educational outcomes. Associations involving general maternal mental health were attenuated after full adjustment.

**Conclusions:**

Persistent maternal psychological distress is associated with poorer child academic outcomes. Although causal inference is not possible, the findings highlight maternal mental wellbeing as an important correlate of child development.

## Introduction

Maternal mental health is increasingly recognized as a fundamental determinant of child development, with consequences that extend beyond infancy into later childhood, adolescence, and educational attainment. Across public health and related disciplines, evidence shows that children of mothers experiencing poor mental health face elevated risks of behavioural difficulties, poorer academic performance, and adverse educational trajectories.^1–4^ These associations are thought to operate through interrelated pathways, including chronic stress exposure, disruptions in parenting practices and emotional availability, and broader socioeconomic disadvantage, positioning maternal mental health as both a health and social policy concern with implications for intergenerational transmission of disadvantage.^5^^,^^6^

A substantial body of evidence from cohort studies and systematic reviews links maternal depression, anxiety, and psychological distress to child outcomes spanning socio-emotional development, behaviour, cognition, and educational achievement.^7–10^ These associations persist across diverse populations and contexts, suggesting that maternal mental health shapes children’s developmental environments over time rather than acting solely through short-lived mechanisms.^11^^,^^12^ Maternal mental health is also embedded within broader family and social environments, where factors such as socioeconomic disadvantage, intimate partner violence, adverse childhood experiences, and neighbourhood conditions may amplify or buffer risks to children.^13^^,^^14^

Despite this extensive literature, several gaps remain. Much existing evidence relies on cross-sectional designs or short follow-up windows, often focusing on pregnancy or the immediate postpartum period. While informative, such approaches provide limited insight into how persistent or cumulative maternal mental health conditions relate to child outcomes across longer developmental horizons.^8^^,^^15^ Emerging evidence suggests that chronic or recurrent maternal distress is more strongly associated with adverse child outcomes than transient episodes, yet relatively few studies capture long-run exposure using repeated measures over time.^11^^,^^12^

In addition, many studies focus on a single domain of child wellbeing rather than adopting a multi-domain perspective encompassing behavioural, academic, and future-oriented educational outcomes. Reliance on single-wave measures of maternal mental health may further obscure the role of sustained exposure by increasing sensitivity to short-term shocks and measurement error.^10^^,^^16^^,^^17^

Longitudinal household panel surveys offer a valuable opportunity to address these limitations. By repeatedly observing maternal mental health over extended periods and linking mothers to their children, such data enable construction of more stable measures that capture cumulative burden and chronicity. Although child development and education outcomes are often collected intermittently due to modular survey designs, these data still permit analysis of meaningful developmental and educational states when combined with richer longitudinal exposure histories.^18^

From a life-course and developmental origins perspective, both the timing and duration of exposure to maternal mental health conditions are likely to matter for child outcomes.^19^ Long-run measures may therefore serve as a useful proxy for cumulative exposure in settings where child outcomes are observed at discrete intervals, while maintaining a population-level, non-causal interpretive focus.^8^^,^^11^^,^^15^

To address these gaps, this study uses nationally representative longitudinal data to examine associations between long-run maternal mental health and multiple dimensions of child outcomes, including behavioural functioning, academic performance, and expectations of future university participation. Maternal mental health is measured using repeated observations across many survey waves, capturing persistent conditions rather than transitory fluctuations. Child outcomes are drawn from dedicated survey modules observed at specific waves. Sequential models with increasing adjustment for child, maternal, and household characteristics are estimated to assess the robustness of associations and the extent to which socioeconomic factors explain observed relationships.

The contribution of this study is four-fold. First, it provides population-level evidence on associations between long-run maternal mental health and multiple child outcomes within a unified framework. Second, it distinguishes between general maternal mental health and psychological distress, highlighting differences in how these dimensions relate to child development. Third, it demonstrates how longitudinal surveys with intermittently observed child outcomes can be leveraged by combining rich exposure histories with modular outcome data. Finally, by adopting a transparent, non-causal interpretive stance, the study contributes descriptive evidence relevant to public health policy, screening strategies, and the design of maternal mental health interventions aimed at improving child development and long-run wellbeing.^20^

## Method

### Study design and data source

This study adopts a longitudinal observational design using nationally representative household panel data to examine associations between maternal mental health and child behavioural, academic, and educational outcomes. The survey follows individuals and families annually, collecting repeated information on mental health, socioeconomic circumstances, and household composition, and enables linkage of mothers and children across waves to examine intergenerational associations. The study is reported in accordance with the Strengthening the Reporting of Observational Studies in Epidemiology guidelines.

A key feature of the data is the modular structure of child questionnaires: detailed child development and education outcomes are collected only in selected waves, whereas maternal mental health is measured repeatedly over a much longer period. The analytic strategy leverages this structure by combining long-run maternal mental health histories with child outcomes observed at discrete developmental stages.

The analytic sample consists of child observations with non-missing outcome measures that can be linked to maternal mental health histories. Sample sizes vary across specifications due to intermittent availability of child modules and item non-response in covariates. All analyses use de-identified unit-record data accessed under licence from the data provider.

### Child outcome measures

Three child outcomes capturing distinct dimensions of development and educational trajectories are examined. Behavioural outcomes are measured using whether the school contacted parents regarding the child’s behaviour (*cebp_final*), coded such that higher values indicate more favourable outcomes. Academic performance (*ceoa_final*) is measured on a five-point ordinal scale, with higher values indicating poorer performance. Expectations regarding future university participation (*cewgu_final*) are measured on a four-point scale, with higher values indicating lower expected likelihood of attendance.

These outcomes are observed in three survey waves corresponding to child development and education modules. Invalid responses are coded as missing. Although the outcomes are binary or ordinal, they are treated as continuous to facilitate comparability across outcomes and specifications, a common approach in population health research when the focus is on the direction and robustness of associations rather than precise marginal effects.

### Maternal mental health measures

Maternal mental health is measured using two indicators derived from repeated observations: a general mental health score (*mh_mean*; range 0–100, higher values indicating better mental health) and psychological distress measured using the Kessler Psychological Distress Scale (*kessler_mean*; higher values indicating greater distress).

Maternal mental health measures were constructed using observations up to and including the survey wave in which the child outcome was measured, avoiding the use of post-outcome information. This approach captures persistent maternal mental health status, reduces measurement error associated with single-wave reports, and aligns with evidence that sustained mental health conditions are more relevant for child outcomes than transitory fluctuations. The resulting measures are time-invariant at the mother level within outcome-specific analyses.

### Covariates

Models adjust sequentially for child, maternal, and household characteristics associated with both maternal mental health and child outcomes. Child-level covariates include age and gender. Maternal and household covariates include maternal education, employment status, household structure, geographic remoteness, and long-term illness. Covariates are measured contemporaneously with the child outcome wave where possible, and invalid responses are coded as missing.

### Empirical specification and estimation

Associations between maternal mental health and child outcomes are examined using linear regression models. Baseline models relate each child outcome to the mother’s long-run mental health measure and are extended sequentially to adjust for child characteristics and maternal and household covariates. Across specifications, coefficients are interpreted as population-level associations conditional on observed characteristics.

The baseline empirical specification is given by:


$$ {Y}_{im}=\alpha +\beta M{H}_m+{\varepsilon}_{im} $$

where *Y_im_* denotes the outcome for child *i* of mother *m*, and *MH_m_* represents the mother’s long-run mental health measure (either general mental health or psychological distress).

All models are estimated using ordinary least squares, with standard errors clustered at the mother level to account for within-family correlation. Fixed-effects estimators are not employed because maternal mental health is operationalized as a time-invariant long-run measure and would be differenced out in such models. The estimation strategy therefore aligns with the study’s estimand: between-family associations between persistent maternal mental health and child outcomes.

### Statistical analysis

The fully adjusted models include a richer set of maternal and household covariates, some of which were not observed in all survey waves. As a result, these models are estimated on a smaller analytic subsample. The primary interpretation of results therefore focuses on consistency of direction and magnitude across model specifications rather than direct comparison of coefficients across columns.

Outcomes are analysed using linear models to facilitate comparability across specifications. Prior research indicates that linear models yield similar substantive conclusions to non-linear alternatives for ordinal outcomes with limited categories.

### Interpretation and ethical considerations

Analyses focus on population-level associations rather than causal effects. Estimates are interpreted descriptively, recognizing the observational nature of the data and the potential role of unobserved factors.

The study uses de-identified unit-record data accessed under licence. All analyses comply with the provider’s confidentiality and data security requirements, and separate ethical approval was not required.

## Results

### Descriptive statistics

Descriptive statistics for all variables used in the analysis are presented in Table 1. The child outcome measures show meaningful variation across behavioural, academic, and educational domains.

**Table 1:** Descriptive statistics of variables used in the analysis.

Panel A: continuous variables					
Variable	*N*	Mean	SD	Min	Max
Behaviour outcomes	47 592	1.843	0.363	1	2
Academic outcomes	47 564	2.354	0.935	1	5
Future university expectations	45 702	2.279	1.044	1	4
Maternal mental health	443 604	71.914	13.559	0	100
Maternal psychological distress	415 543	16.686	5.828	10	50
Age (years)	1 364 583	36.586	22.851	0	103
Maternal education	1 012 398	5.981	2.686	1	9
Household structure	1 312 842	5.415	2.910	1	10
Household remoteness	1 364 268	0.542	0.781	0	4
*Panel B: binary and categorical variables (%)*					
*Variable*	*N*	*Category*	*%*		
Gender	1 364 583	Female	51.30		
		Male	48.70		
Employment status	642 087	Employee	84.54		
		Employee of own business	5.43		
		Employer/self-employed	9.61		
		Unpaid family worker	0.42		
Long-term illness	1 360 515	Yes	20.08		
		No	79.92		

This table presents descriptive statistics for all variables used in the regression analyses. Panel A reports mean, standard deviations (SD), and ranges for continuous variables. Panel B reports percentages for binary and categorical variables.

Behavioural outcomes indicate whether the child’s school contacted parents regarding poor behaviour, where higher values represent more favourable outcomes. Academic performance is measured on a five-point scale, with higher values indicating poorer performance. Future university expectations are measured on a four-point scale, with higher values indicating a lower expected likelihood of university attendance.

Maternal mental health is measured on a 0–100 scale, with higher values indicating better mental health. Maternal psychological distress is measured using the Kessler Psychological Distress Scale, with higher values indicating greater distress. Maternal education is measured on a nine-point scale. Household structure and household remoteness are coded such that higher values reflect greater household complexity and remoteness, respectively.

Sample sizes vary across variables due to item non-response and differences in questionnaire modules across survey waves.

As shown in Table 1, school-reported behavioural outcomes (*cebp_final*) have a mean of 1.84 (SD = 0.36) on a two-point scale, indicating that most children did not experience behavioural problems, with limited dispersion. Academic performance (*ceoa_final*) exhibits greater variability, with a mean of 2.35 (SD = 0.94) on a five-point scale, reflecting heterogeneity in school performance. Expectations regarding future university attendance (*cewgu_final*) have a mean of 2.28 (SD = 1.04), indicating substantial variation in anticipated educational trajectories.

Maternal mental health (*mh_mean*) averages 71.9 (SD = 13.6) on a 0–100 scale, suggesting moderate overall wellbeing with considerable variation across mothers. Maternal psychological distress (*kessler_mean*) has a mean of 16.7 (SD = 5.8), spanning a wide range of values.

The average age in the pooled analytic sample is 36.6 years (SD = 22.9). Maternal education averages 6.0 (SD = 2.7) on a nine-point scale. Approximately 51.3% of observations are female, 84.5% of mothers are employed, and 20.1% report a long-term illness. Overall, the descriptive statistics indicate sufficient variation across exposures, outcomes, and covariates to support multivariable regression analysis.

### Main regression results

Table 2 reports associations between maternal mental health and child outcomes across three specifications: unadjusted models, models adjusted for child age and gender, and fully adjusted models that additionally control for maternal education, employment status, household disadvantage, housing stress, geographic remoteness, and long-term illness. All models are estimated using ordinary least squares with standard errors clustered at the mother level. Sample sizes decline in the fully adjusted models due to the inclusion of covariates with intermittent availability; however, the direction of associations remains broadly consistent with simpler specifications.

**Table 2:** Maternal mental health and child outcomes.

Panel A. Behavioural problems			
	(1) Unadjusted	(2) + Child controls	(3) + Full controls
Maternal mental health (long-run average)	0.0032^***^	0.0033^***^	0.0012
	(0.0004)	(0.0004)	(0.0008)
Maternal psychological distress (Kessler scale)	−0.0083^***^	−0.0084^***^	−0.0034^*^
	(0.0011)	(0.0011)	(0.0019)
Observations	26,308	26,308	2789
Clusters (mothers)	3491	3491	1070
*R* ^2^	0.013	0.018	0.010
*Panel B. School performance*			
	*(1) Unadjusted*	*(2) + Child controls*	*(3) + Full controls*
Maternal mental health (long-run average)	−0.0090^***^	−0.0091^***^	−0.0055^**^
	(0.0012)	(0.0012)	(0.0021)
Maternal psychological distress (Kessler scale)	0.0211^***^	0.0214***	0.0119^**^
	(0.0028)	(0.0028)	(0.0051)
Observations	26 300	26 300	2789
Clusters (mothers)	3493	3493	1071
*R* ^2^	0.015	0.018	0.027
*Panel C. Educational expectations*			
	*(1) Unadjusted*	*(2) + Child controls*	*(3) + Full controls*
Maternal mental health (long-run average)	−0.0079^***^	−0.0081^***^	−0.0043
	(0.0014)	(0.0014)	(0.0027)
Maternal psychological distress (Kessler scale)	0.0202^***^	0.0205^***^	0.0122^*^
	(0.0034)	(0.0034)	(0.0065)
Observations	25 281	25 281	2704
Clusters (mothers)	3430	3430	1059
*R* ^2^	0.009	0.015	0.078

Each column reports coefficients from ordinary least squares regressions estimating associations between maternal mental health and child outcomes. Panel A reports behavioural outcomes (school contact due to child behaviour), where higher values indicate more favourable outcomes. Panel B reports academic performance measured on a five-point scale, where higher values indicate poorer performance. Panel C reports expectations of future university participation measured on a four-point scale, where higher values indicate a lower expected likelihood of attendance.

Maternal mental health is measured using a general mental health score (range 0–100, higher values indicate better mental health) and maternal psychological distress is measured using the Kessler Psychological Distress Scale (higher values indicate greater distress). Column (1) reports unadjusted models; column (2) adjusts for child age and gender; column (3) additionally adjusts for maternal education, employment status, household structure, geographic remoteness, and long-term illness. Standard errors clustered at the mother level are reported in parentheses.

^*^ *P* < .05.

^**^ *P* < .10.

^***^ *P* < .01.

### Behavioural outcomes

In unadjusted and child-adjusted models, higher maternal mental health scores are associated with more favourable child behavioural outcomes (β = 0.0032–0.0033, *P* < .01). This association is attenuated and no longer statistically significant after full adjustment for maternal and household characteristics (β = 0.0012, SE = 0.0008).

In contrast, higher maternal psychological distress is associated with less favourable behavioural outcomes. Although attenuated after full adjustment, the association remains negative and marginally statistically significant (β = −0.0034, SE = 0.0019, p < 0.10).

### Academic outcomes

Higher maternal mental health is associated with more favourable academic performance across specifications. In fully adjusted models, higher maternal mental health scores are associated with lower values on the academic outcome scale (β = −0.0055, SE = 0.0021, *P* < .05), indicating better academic performance.

Maternal psychological distress is consistently associated with poorer academic outcomes. In fully adjusted models, a one-unit increase in psychological distress is associated with poorer academic performance (β = 0.0119, SE = 0.0051, *P* < .05).

### Future university attendance outcomes

Higher maternal mental health is associated with more favourable expectations regarding future university participation in unadjusted and child-adjusted models; however, this association is attenuated and no longer statistically significant after full adjustment for maternal and household characteristics (β = −0.0043, SE = 0.0027).

In contrast, maternal psychological distress remains positively associated with poorer expectations regarding university participation. In fully adjusted models, a one-unit increase in maternal psychological distress is associated with a higher likelihood of unfavourable university expectations (β = 0.0122, SE = 0.0065, *P* < .10).

### Robustness and supplementary analyses

Results from specifications focusing exclusively on maternal psychological distress are also reported in Table 2. These estimates are consistent in direction and relative magnitude with those reported in the main analysis.

Appendix Table A1 examines heterogeneity by childbirth order. For most outcomes, estimated associations are similar across first-, second-, and third-born children, and formal tests do not indicate statistically significant differences. An exception is observed for behavioural outcomes associated with maternal mental health, where modest heterogeneity is detected (*P* = .041), with slightly larger associations among first-born children.

**Figure 1:**
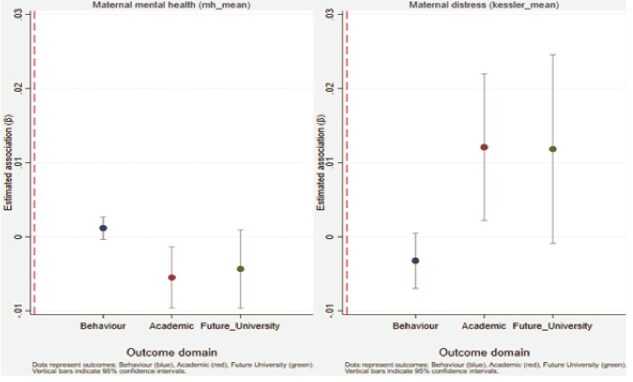
Associations between maternal mental health and child outcomes notes: This figure presents fully adjusted regression coefficients (β) and 95% confidence intervals for associations between maternal mental health and child outcomes across three domains: Behavioural outcomes, academic performance, and expectations regarding future university participation. The left panel shows associations with maternal mental health, where higher values indicate better mental health. The right panel shows associations with maternal psychological distress, where higher values indicate greater distress. Dots represent point estimates, and vertical bars indicate 95% confidence intervals. The three outcome domains are presented seperately within each panel. All estimates are derived from ordinary least squares models adjusted for child age and gender, maternal education, employment status, household structure, geographic remoteness, and long-term illness, with standard errors clustered at the mother level. Positive coefficients indicate more favourable outcomes for maternal health and less favourable outcome for maternal distress.

Figure 1 provides a visual summary of fully adjusted associations between maternal mental health, maternal psychological distress, and child outcomes. Associations involving maternal psychological distress are generally larger in magnitude and more consistently distinguishable from zero, particularly for academic outcomes, than those for general maternal mental health.

### Summary of results

Across behavioural, academic, and educational domains, maternal mental health and maternal psychological distress are associated with child outcomes in unadjusted and partially adjusted models. Adjustment for maternal and household characteristics attenuates several associations. However, associations involving maternal psychological distress, particularly for academic outcomes, remain evident after full adjustment.

## Discussion

### Main finding of this study

Using nationally representative longitudinal data, this study documents population-level associations between maternal mental health and child outcomes across behavioural, academic, and educational domains. In unadjusted and child-adjusted models, better maternal mental health is associated with more favourable child outcomes, while higher maternal psychological distress is associated with poorer outcomes across school-reported behavioural problems, academic performance, and expectations of future university participation.

After adjustment for maternal and household characteristics, several associations are attenuated, indicating that socioeconomic conditions and family context explain part of the observed relationships. However, associations involving maternal psychological distress persist in fully adjusted models, particularly for academic performance and, to a lesser extent, educational expectations. Although modest at the individual level, these associations are meaningful in population terms given the prevalence of maternal psychological distress and the cumulative nature of educational disadvantage.

### What is already known on this topic

A substantial body of public health and interdisciplinary research has established that maternal mental health is associated with a wide range of child outcomes, including behavioural difficulties, emotional problems, cognitive development, and educational achievement. Evidence from cohort studies and systematic reviews consistently shows that maternal depression, anxiety, and psychological distress are linked to adverse child outcomes across diverse populations and settings, with effects often extending beyond early childhood.

Prior research also suggests that chronic or recurrent maternal distress may be more strongly associated with adverse child outcomes than transient episodes. However, much of the existing literature relies on cross-sectional designs, short follow-up periods, or single-wave measures of maternal mental health, limiting insight into how sustained maternal mental health conditions relate to child outcomes across longer developmental horizons. Many studies further focus on single domains of child wellbeing, constraining understanding of how maternal mental health may shape multiple, interconnected aspects of child development.

### What this study adds

This study contributes to the literature in several ways. First, it provides population-level evidence on associations between long-run maternal mental health and multiple child outcomes within a unified framework, addressing limitations of short-run and single-domain analyses. By constructing maternal mental health measures from repeated observations, the analysis captures persistent exposure and reduces reliance on single-wave reports. The findings highlight the public health relevance of persistent maternal psychological distress for children’s educational trajectories, even when child outcomes are measured intermittently.

Second, the study distinguishes between general maternal mental health and psychological distress, showing that distress-based measures display more consistent associations with children’s academic and educational outcomes after extensive adjustment. Third, it demonstrates how longitudinal household panel data with intermittently observed child outcomes can be leveraged by combining rich exposure histories with outcomes measured at key developmental stages. Finally, by adopting a transparent, non-causal interpretive stance, the findings contribute descriptive evidence relevant to public health policy, screening strategies, and the design of maternal mental health interventions.

### Limitations of this study

Several limitations should be acknowledged. First, child outcome measures are available in a limited number of survey waves due to the modular survey design. However, these outcomes capture relatively stable behavioural and educational states, while maternal mental health is observed repeatedly over a longer period, enabling the construction of robust long-run exposure measures.

Second, despite adjustment for a wide range of child, maternal, and household characteristics, unobserved factors, such as parenting practices, family relationships, or genetic endowments, may contribute to the observed associations. Accordingly, the findings should not be interpreted as causal effects. Third, child outcomes are treated as continuous measures for comparability across domains, abstracting from their underlying categorical nature. While this approach may attenuate effect sizes, it is unlikely to alter the direction of observed associations.

Fourth, sample sizes decline in fully adjusted models due to item non-response in maternal socioeconomic variables. While this may limit generalizability, the sequential modelling strategy allows transparent assessment of estimate sensitivity, and the consistency of findings across less adjusted specifications suggests that results are not driven solely by sample selection. Finally, although maternal mental health is measured contemporaneously with some child outcomes, the analysis does not attempt causal inference, and results should be interpreted as associations.

## Conclusion

This study provides population-level evidence of associations between maternal mental health and children’s behavioural, academic, and educational outcomes using nationally representative longitudinal data. Associations involving maternal psychological distress persist after extensive adjustment, particularly for academic outcomes.

By combining long-run measures of maternal mental health with intermittently observed child outcomes, the analysis underscores maternal mental health as an important public health concern with potential implications for child development and educational inequality. These findings support the value of policies and interventions that prioritize maternal mental wellbeing as part of broader investments in child and family health.

## Supplementary Material

Supplementary_Material_fdag032

## Data Availability

The data used in this study are not publicly available due to licensing restrictions but can be accessed by eligible researchers through application to the data provider. Analytical code is available from the corresponding author on reasonable request.

## Notes

**Minnat Seema,** PhD candidate

**Clifford Afoakwah,** Senior Research Fellow

**Joshua Byrnes,** Professor
