# Supplementary material for: Persistent maternal mental health and child’s behavioural, academic, and educational outcomes: evidence from national longitudinal study
Source: J Public Health (Oxf). 2026 Apr 25;48(2):421–9. doi: 10.1093/pubmed/fdag032 (PMC13223587; doi:10.1093/pubmed/fdag032)
Supplement: Supplementary_Material_fdag032 [file supplementary_material_fdag032.zip › Appendix_Table_A1_fdag032.docx]

| **Appendix Table A1. Heterogeneity in Associations Between Maternal Mental Health and Child Outcomes by Birth Order** | | | | |
| --- | --- | --- | --- | --- |
| **Panel A: Maternal mental health** | | | | |
| **Outcome** | **1st child** | **2nd child** | **3rd child** | **p-value (heterogeneity)** |
| **School performance** | −0.0093*** | −0.0092*** | −0.0086*** | 0.945 |
| **Behavioural problems** | 0.0041*** | 0.0028*** | 0.0021** | **0.041** |
| **Educational expectations** | −0.0086*** | −0.0077*** | −0.0077** | 0.888 |
| **Panel B: Maternal psychological distress** | | | | |
| **Outcome** | **1st child** | **2nd child** | **3rd child** | **p-value (heterogeneity)** |
| **School performance** | 0.0213*** | 0.0215*** | 0.0216*** | 0.998 |
| **Behavioural problems** | −0.0098*** | −0.0074*** | −0.0067** | 0.229 |
| **Educational expectations** | 0.0220*** | 0.0195*** | 0.0190** | 0.823 |
| **Notes:** Table reports coefficients from linear regression models interacting maternal mental health with child birth order. All models control for child age and gender and include survey wave fixed effects. Standard errors are clustered at the maternal level. P-values in the final column report joint F-tests of equality of coefficients across birth-order groups. ***p < 0.01, **p < 0.05, *p < 0.10. | | | | |
