# Supplementary material for: Persistent maternal mental health and child’s behavioural, academic, and educational outcomes: evidence from national longitudinal study
Source: J Public Health (Oxf). 2026 Apr 25;48(2):421–9. doi: 10.1093/pubmed/fdag032 (PMC13223587; doi:10.1093/pubmed/fdag032)
Supplement: Supplementary_Material_fdag032 [file supplementary_material_fdag032.zip › STROBE_Persistent_Maternal_Mental_Health_and_Child_Behavioural_Academic_and_Educational_Outcomes_fdag032.docx]

STROBE Statement—Checklist of items that should be included in reports of ***cohort studies***

**Title: Persistent Maternal Mental Health and Child’s Behavioural, Academic and Educational Outcomes: Evidence from National Longitudinal Study**

|  | Item No | Recommendation |
| --- | --- | --- |
| **Title and abstract** | 1 | (*a*) The title and abstract describe the study as a nationally representative longitudinal observational study using household panel data. |
|  |  | (*b*) The abstract provides a balanced summary of the data source, study population (linked mother–child observations), exposures, outcomes, statistical methods (ordinary least squares regression with standard errors clustered at the mother level), and the direction of key findings. |
| Introduction | | |
| Background/rationale | 2 | The introduction describes the scientific background linking maternal mental health to child behavioural, academic, and educational outcomes, highlights relevance to population health and intergenerational disadvantage, and identifies gaps related to long-run exposure measurement and intermittently observed child outcomes. |
| Objectives | 3 | The study objectives are to examine associations between long-run maternal mental health and child behavioural, academic, and educational outcomes using longitudinal data, and to assess robustness across sequentially adjusted models. |
| Methods | | |
| Study design | 4 | This study is an observational longitudinal cohort analysis using secondary data from the Household, Income and Labour Dynamics in Australia (HILDA) Survey. |
| Setting | 5 | The study setting is Australia, using nationally representative household survey data collected annually between 2001 and 2022 with repeated follow-up of participants across waves. |
| Participants | 6 | (*a*) Eligible participants included children with valid behavioural, academic, and educational outcome measures who could be linked to their biological mothers using survey identifiers. |
|  |  | (*b*) Not applicable; this study did not use matching. |
| Variables | 7 | Maternal mental health measures were the primary exposures. Child outcomes included behavioural functioning, academic performance, and expectations of future university participation. Covariates included child age and gender, maternal education, employment status, household characteristics, geographic remoteness, and long-term illness. |
| Data sources/ measurement | 8* | All variables were derived from validated HILDA Survey instruments, including SF-36 mental health items and the Kessler psychological distress scale. Mother–child linkages were established using HILDA genealogical identifiers, with consistent measurement methods across survey waves*.* |
| Bias | 9 | Potential sources of bias were addressed through multivariable adjustment for confounding, exclusion of invalid response categories, use of long-run exposure measures to reduce measurement error, and clustering of standard errors at the mother level, while acknowledging potential residual confounding. |
| Study size | 10 | The study size was determined by the number of linked mother–child observations meeting inclusion criteria after data cleaning and availability of valid outcome, exposure, and covariate information. |
| Quantitative variables | 11 | Maternal mental health measures were analysed on their original scales as long-run averages. Child outcome measures were analysed as continuous variables to facilitate comparability across outcomes. Age was modelled flexibly using linear and quadratic terms. |
| Statistical methods | 12 | (*a*) Associations were estimated using linear regression models estimated by ordinary least squares, with standard errors clustered at the mother level to account for within-family correlation.” |
|  |  | (*b*) Observations with invalid or non-response codes were excluded. Analyses were conducted using complete cases for each model, and the resulting sample sizes are reported accordingly. |
|  |  | (*c*) Observations with invalid or non-response codes were excluded. Analyses were conducted using complete cases for each model, and sample sizes are reported accordingly. |
|  |  | (*d*) The HILDA Survey is an unbalanced longitudinal panel in which participants may enter and exit across waves. This structure was accommodated through clustered standard errors and model-specific sample definitions; no explicit attrition modelling was undertaken. |
|  |  | (*e*) Sensitivity analyses included alternative mental health outcome measures and model specifications with and without covariate adjustment. |
| Results | | |
| Participants | 13* | (a) The number of observations at each stage of data cleaning and analysis is reported, including the final analytic sample for each outcome |
|  |  | (b) Reasons for exclusion included missing linkage, invalid survey responses, and missing outcome or exposure data. |
|  |  | (c) A flow diagram was not included due to the use of secondary longitudinal survey data with repeated observations across waves. |
| Descriptive data | 14* | (a) Descriptive characteristics of children and mothers, including health measures and socioeconomic variables, are presented. |
|  |  | (b) The number of observations available for each variable is reported in descriptive tables and model sample sizes. |
|  |  | (c) Participants contributed repeated observations across multiple survey waves, reflecting longitudinal follow-up. |
| Outcome data | 15* | Summary statistics for child behavioural, academic, and educational outcomes are reported using descriptive tables and model-specific sample sizes. |
| Main results | 16 | (*a*) Unadjusted and adjusted regression estimates are presented with 95% confidence intervals, with confounders specified. |
|  |  | (*b*) Not applicable; continuous variables were analysed without categorisation. |
|  |  | (*c*) Outcomes were analysed as continuous measures to facilitate comparability across behavioural, academic, and educational outcomes. |
| Other analyses | 17 | Additional analyses examined robustness across alternative maternal mental health measures and model specifications. |
| Discussion | | |
| Key results | 18 | Key findings are summarised in relation to the study objectives. |
| Limitations | 19 | Study limitations, including residual confounding, self-reported measures, and intermittent availability of child outcomes due to the modular survey design, are discussed. |
| Interpretation | 20 | Results are interpreted cautiously in light of the study design, existing literature, and implications for population mental health, quality of life, and preventive public health strategies |
| Generalisability | 21 | Findings are generalisable to the Australian household population, with limitations for non-household and institutionalised populations. |
| Other information | | |
| Funding | 22 | This study did not receive any specific external funding. |

*Give information separately for exposed and unexposed groups.

**Note:** An Explanation and Elaboration article discusses each checklist item and gives methodological background and published examples of transparent reporting. The STROBE checklist is best used in conjunction with this article (freely available on the Web sites of PLoS Medicine at http://www.plosmedicine.org/, Annals of Internal Medicine at http://www.annals.org/, and Epidemiology at http://www.epidem.com/). Information on the STROBE Initiative is available at http://www.strobe-statement.org.
